# A Retrospective Study on the Transferring Accuracy of a Fully Guided Digital Template in the Anterior Zone

**DOI:** 10.3390/ma14164631

**Published:** 2021-08-17

**Authors:** Lirong Huang, Xiaoqing Zhang, Anchun Mo

**Affiliations:** Department of Implant Dentistry, West China Hospital of Stomatology, Sichuan University, Chengdu 610041, China; lirong_huang@stu.scu.edu.cn (L.H.); 2017224035151@stu.scu.edu.cn (X.Z.)

**Keywords:** anterior zone, fully guided template, digital implantology, accuracy deviation

## Abstract

The accuracy of implant placement with a fully guided digital template can be influenced by many factors, such as arch difference, alveolar bone density, timing of implant placement and open flap. The purpose of this article was to evaluate the factors presumptively affecting the accuracy of implant placement assisted by the fully guided template in the anterior zone. In 40 patients with missing anterior teeth, a total of 52 implants were placed with tooth-borne, fully guided templates after CBCT evaluation, in West China Hospital of Stomatology, Sichuan University. After overlapping the pre-and post-operative DICOM data, measurements were taken in the dental implant planning software (Nobel Clinician^®^) to calculate linear and angular deviations between virtual placement plan and actual implant placement. Grouping was categorized according to three factors that possibly have an influence on accuracy: arch type (maxilla/mandible), timing of implant placement (immediate/delayed), surgical technique (open flap/flapless). The data was analyzed with independent sample *t*-test (*p* < 0.05). The results showed that the apical, coronal, depth and angular mean deviations of implant positions in anterior zone were 1.13 ± 0.39 mm, 0.86 ± 0.33 mm, 0.41 ± 0.66 mm, 3.32 ± 1.65° with the fully guided templates. The accuracy at apex level, coronal level and the angulation were similar between the maxilla and mandible, and the magnitude of all four deviations between immediate and delayed implantation, open flap and flapless technique were small. No statistically significant difference was observed (*p* > 0.05). Whereas there was significant difference in depth deviation between maxilla and mandible (*p* < 0.05). Conclusively, the implant site, alveolar bone density, timing of implant placement and surgical techniques merely compromise the implant placement accuracy under the assistance of a fully guided template.

## 1. Introduction

Implant restorations in the anterior zone are usually accompanied with patients’ higher aesthetic expectations and sufficient available bone volume. Slight deviations of the implant position may harm important anatomical structures such as adjacent teeth, or lead to a series of esthetic, biological and technical complications [1,2]. Therefore, how to avoid the occurrence of adverse events and achieve the precise placement of the optimal three-dimensional position of the implant is a challenge for every implantologist.

The wide usage of CBCT data in combination with implant planning software has made it possible to lead predictable outcomes. One possible technique that facilitates a more accurate implant positioning is computer-assisted, template-guided implantology [3,4]. Different from the traditional free-hand implant placement that overly relies on the clinician’s skill set and experience, computer-assisted, template-guided implantology is driven by final optimal restoration, according to which clinicians address considerations for the most optimal implant position prior to the surgery [5,6,7]. The template has been postulated to provide higher precision [8,9,10,11], which can help to achieve better implant placement with the potential for reduced operative complications [12,13,14]. Through digital designing software such as Simplant^®^ and Nobel Clinician^®^, the patient’s cone-beam computed tomography (CBCT) data was overlapping with the intraoral or model three-dimensional data obtained by an optical scanner [15], so that the clinicians can virtually plan the optimal three-dimensional implant position before the operation. Then, using the computer-aided design and computer-aided manufacturing (CAD-CAM) technology to fabricate the surgical template, and with the assistance of which we can transfer the preoperative virtual plan into the actual surgery.

According to the surgical template and its effects on the accuracy of the surgical protocols, template guided surgery could principally be differentiated into fully guided and pilot-drill-guided protocols [10]. Fully guided surgery is facilitated with a particular drill kit, inserting different drills through guiding sleeves in the template step by step, until the final insertion of the implant fixture. A pilot-drill-guided protocol is performed with a universal guide kit. After the pilot drill with the assistance of the template, with or without a drill stop ring, the template is removed, and subsequent drilling is performed by free-hand. Extensive research has shown that, compared with pilot-drill-guided surgery, fully guided protocol can better control the linear and angular deviations, and enables more accurate implant placement [10,13,16,17]. Nowadays, fully guided surgical templates are widely used in various surgeries for the purpose of improving surgical precision and safety. To date, however, most relevant research mainly focuses on the edentulous jaw and posterior zone [17,18,19]; there have been very few discussions on the accuracy of anterior implant placement with fully guided surgery, and further verifications are still needed.

There are various factors that affect the accuracy of the guided template. In the entire process of computer-assisted, template-guided implant surgery, from preoperative data collection, data overlap, guided template production, to the operation, multiple links may have impact on the accuracy of implantation. The accumulation of a little error in each link will eventually lead to the significant positional deviation of the implant [20]. Several studies have pointed out that bone density has a certain influence on the angle of implant placement. Lower bone density may cause greater implant angular deviation [21]. In terms of bone density, the maxillary bone density is generally lower than that of the mandible. Yet, when a fully guided template is utilized, whether or not the disparities would still exist, leads to higher deviations in the maxillary guided surgery, or bias may be reduced due to its higher transferring accuracy. Further research on this would be valuable.

In order to reduce surgical interventions and shorten the treatment period [22,23,24], immediate implantations are often applied in the anterior zone. Unlike the preparation of conventional implantation, in the implant site preparation after tooth extraction, the drill often drifts labially due to the resistance of the lingual bone wall of the extraction socket, which cause deviations in the final implant position.

To reduce the adverse impact of conventional open flap surgery, given sufficient vertical height, width and bone density of the alveolar ridge as well as adequate width and thickness of keratinized gingiva, fully guided flapless protocol can be conducted in the anterior zone. Schnutenhaus et al. [25] found no statistical significance of the effect on implantation accuracy between open flap and flapless techniques. Behneke et al. [26] pointed out that the implant position is shallow when the flap is not elevated. Hence, more studies related to discrepancies of the implant placement in the anterior zone with or without open flap techniques with tooth-borne surgical template need to be initiated.

In this study, a digital method is adopted to measure the transferring accuracy of the surgical template in the anterior zone. We can also clarify the influence among the arch difference, timing of implant placement and surgical techniques by comparing the accuracy of the fully guided template between the maxilla and mandible groups, immediate and delayed implantation groups, open flap and flapless technique groups. It aims to provide a theoretical basis and practical guidance for the application of the fully guided implantation.

## 2. Materials and Methods

### 2.1. Patient Selection

Forty patients (aged 18–75 years) who received fully guided surgery in the anterior zone (upper and lower anterior teeth) were recruited in West China Hospital of Stomatology, Sichuan University, from January 2019 to April 2021. Patients were screened according to the following criteria:

Inclusion criteria:

1. Age from 18 to 75 years old;
2. The anterior teeth were missing or need to be extracted, requiring implant surgery;
3. Sufficient teeth to support a tooth-supported template;
4. In good health and can tolerate implant surgery;
5. Fully understand the surgical plan and sign an informed consent form voluntarily.

Exclusion criteria:

1. Uncontrolled infection and inflammation at the implant site
2. Uncontrolled systemic diseases
3. Pregnancy or lactation
4. History of local irradiation therapy;
5. Psychiatric problems;
6. Alcohol, tobacco (>10 cigarettes per day) or drug abuse;
7. Severe bruxism or clenching;
8. Poor oral hygiene habits and poor compliance.

The study was conducted in accordance with the Helsinki Declaration of 1975, as revised in 2013, and has been approved by the Ethics Committee of West China Hospital of Stomatology, Sichuan University (Approval NO. WCHSIRB-D-2017-113).

### 2.2. Preoperative Preparation

Before the operation, wide-field cone beam computed tomography (CBCT) scans (3D Accuitomos^®^, Morita, Tokyo, Japan) were taken of all patients, to obtain the bone tissue information (the specific shooting parameters of CBCT are as follows: tube current: 5 mA; tube voltage: 90 kv; shooting time: 17.5 s; voxel: 0.25 mm; layer thickness: 0.25 mm; field of view: 140 mm × 100 mm), then export and save the data in DICOM format. The intraoral or plaster model three-dimensional data were also needed and obtained by optical scanner (Carestream Dental CS3600^®^, Straumman, Basel, Switzerland). Import the scanning data to the dental designing software (3 Shape Dental System^®^, 3 Shape, København, Denmark) and conduct the virtual prosthesis design, and export the data into surface tessellation language (STL) format. 

Reconstruct the maxilla from DICOM data and overlap it with STL data through the shared tooth information in the NobelClinician^®^ digital design software. The optimal three-dimensional position of the implant was planned to follow the “restoration-oriented” principle and ensure a sufficient safety distance between the implant and the adjacent teeth and the implant. Add a guided anchor pin in the appropriate position to increase the stability of the tooth-borne surgical template. Finally, generate the surgical template abide by the procedure of the software. After confirmation, it will be sent to the prothesis processing factory and fabricated.

### 2.3. Surgical Procedure

**Figure 1:**
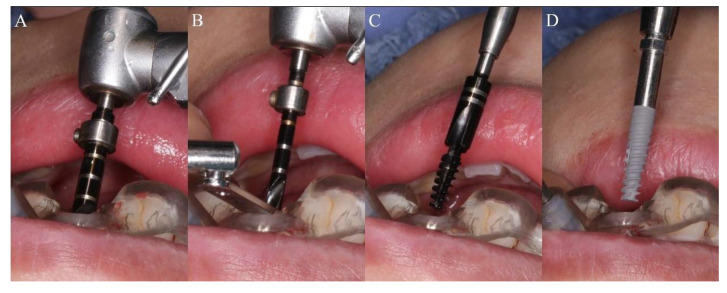
The sequence of the drill in immediate implantation protocol: (**A**) Remove the resistance of the lingual/palatal bone wall with a large-diameter guided twist drill; (**B**) Preparation with sequential guided twist drills and corresponding guided drill guides under the guidance of the template; (**C**) Tapping with guided screw tap; (**D**) Implant insertion.

**Figure 2:**
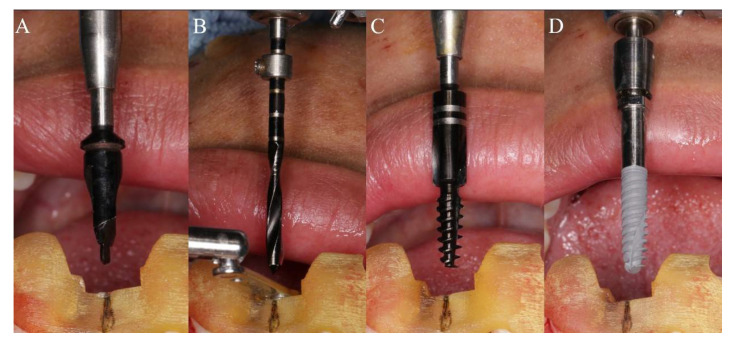
The sequence of the drill in conventional implantation protocol: (**A**) Conform the position with the initial guide drill under the guidance of the surgical template; (**B**) Preparation with sequential guided twist drills and corresponding guided drill guides under the guidance of the template; (**C**) Tapping with guided screw tap; (**D**) Implant insertion.

All operations were performed by the same implant doctor with extensive clinical experience, immersed in iodophor and disinfected using the surgical template for 30 min. The patient took a supine position, routinely disinfected and draped. Used 4% articaine to perform local infiltration anesthesia on the operation area. After the anesthesia being onset of effect, extracted the hopeless tooth or took an open flap technique in the operation area. Then, worn the surgical template throughout the whole process, and checked the fit of the template through the inspection window. Finally, fixated it with a guided anchor pin. The fully guided surgery from the initial drill to the final insertion was conducted by NobelActive^®^Guide Drilling Kit (Nobel Biocare AB, Sweden) with a surgical template. Different preparation methods were used for immediate implantation and delayed implantation. During immediate implantation (Figure 1A–D), a large-diameter guided twist drill was applied for initial preparation. The drill was only inserted into a certain depth to remove the resistance of the lingual/palatal bone wall, to reduce the occurrence of the slipping and drifting of the subsequent drills. Then, the drilling was performed using sequential drills with increasing diameter and corresponding guided drill guides under the guidance of the template, according to the manufacturer’s instructions. If necessary, a guided screw tap would be used for half or the whole process according to the bone density. Finally, the implant was placed. In order to reduce the deviations of implant placement in immediate implantation, we conducted some adjustments and changes in the surgical method. After the guided template is retained, a large-diameter drill close to the implant size is firstly applied in the initial stage of preparation to eliminate the obstacle of lingual side and shape a platform with a certain width. In this way, the contact surface of the subsequent drills with bone wall is no longer a slope during preparation, and the drilling can be more stable, allowing more precise implantation under the guidance of the guided template throughout the entire process. During delayed implantation (Figure 2A–D), the initial guide drill is used to conform the position under the guidance of the surgical template, and then applied the guided twist drills, step by step. If necessary, a guided screw tap would be used for half or the whole process according to the bone density. Finally, the implant is inserted. The stop rings on the guided twist drills and the guided drill could effectively control the depth of preparation and implantation. When the initial stability of the implant reached 35 N·cm, it could be immediately loaded with temporary prosthesis, otherwise the cover screw was used for embedded healing.

### 2.4. Bone Density Measurement

**Figure 3:**
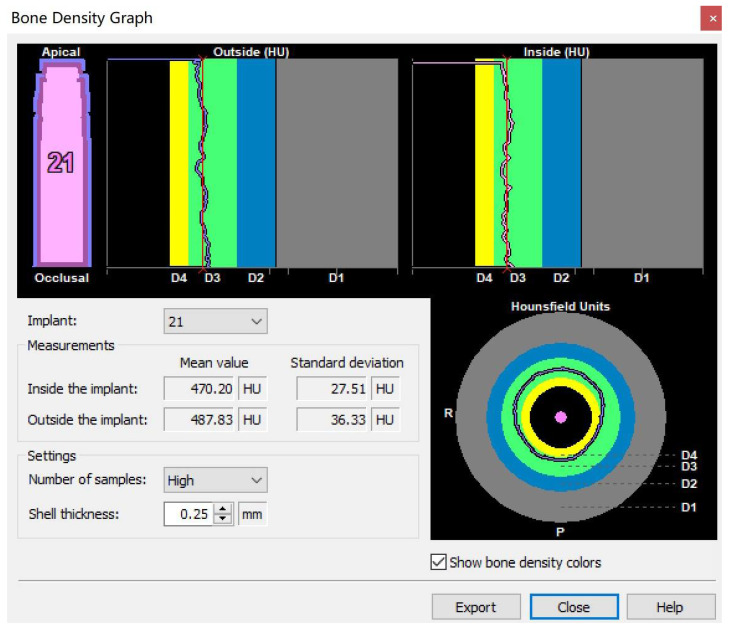
The bone density graph: The shell thickness was set to be 0.25 mm, and the number of samples was high. The mean Hounsfield unit (HU) value of outside the implant could be taken for the bone density.

Import the preoperative DICOM data into Simplant17.0 software, plan the implant in the ideal position and use the bone density around implant tool to obtain a bone density graph (Figure 3). The shell thickness was set to be 0.25 mm, and the number of samples were high. The mean Hounsfield unit (HU) value of outside the implant could be taken for the bone density.

### 2.5. Accuracy Measurement

**Figure 4:**
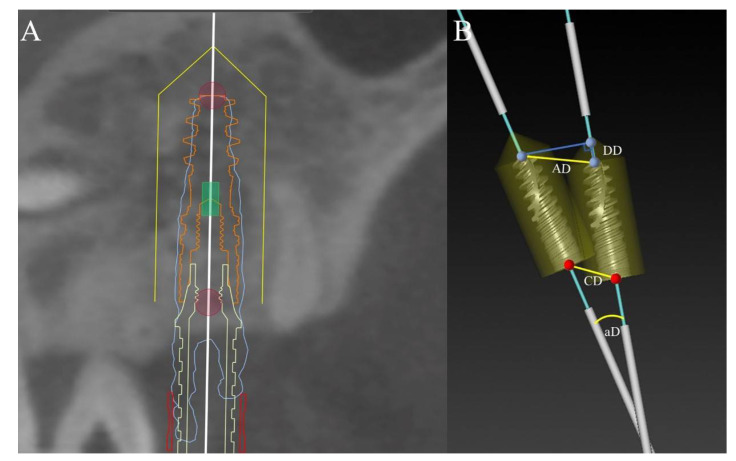
Accuracy measurement: (**A**) Registration according to the contour of the real implant position with a new implant; (**B**) The measurement of coronal deviation (CD), apical deviation (AD), depth deviation (DD) and angular deviation (aD).

Import the postoperative DICOM data into the Mimics software (Mimics^®^, Materialise, Leuven, Belgium) for 3D model reconstruction. The reconstructed model contains the 3D contour of the implant and the information of the adjacent teeth and jaw. Saved it as STL format and import it into Nobel Clinician^®^ software. Then overlapped it with the corresponding preoperative design data. According to the contour of the real implant position, a new implant is placed for registration (Figure 4A). Blinding was set for outcome assessors and the trial statistician. Measured the coronal deviation (CD), apical deviation (AD), depth deviation (DD) and angular deviation (aD) between the virtual and real positions of the implant (Figure 4B). The methods to measure each index were as follows:

1. The coronal deviation (CD) is the linear distance of coronal centers between the two implants;
2. The apical deviation (AD) is the linear distance of apical centers between the two implants;
3. The depth deviation (DD) is the vertical distance of apical centers between the two implants. The positive value meant the actual position is deeper than the virtual position, otherwise the negative value meant the actual position is higher than the virtual position;
4. The angular deviation (aD) is the angle formed by the long axes of the two implants;

The data were measured by the same surveyor taking the average of 3 measurements.

### 2.6. Statistical Analysis

All data were presented using descriptive statistics; for example, mean, standard deviation (SD), minimum, and maximum. The Shapiro–Wilk test revealed that the data followed a normal distribution (*p* > 0.05). So, independent-samples t-test was used to compare deviation parameters between the maxilla and mandible groups, immediate and delayed implantation groups, open flap and flapless technique groups. Pearson product-moment correlation coefficient and Spearman’s rank correlation coefficient were used to determine the relationships between the deviation values and the bone density. The statistical evaluation was performed using SPSS software (SPSS Version 25.0; SPSS, Chicago, IL, USA) at a significance level of *p* < 0.05.

## 3. Results

### 3.1. Characteristics of the Patients

Forty patients were enrolled in this study (The data has been attached in Supplementary Materials). Because 12 of the 40 patients were inserted with 2 implants, a total of fifty-two implants were measured and statistically analyzed in this study. According to the arch difference, the timing of implant placement and surgical techniques, the patients were divided into maxilla and mandible groups, immediate and delayed implantation groups, open flap and flapless technique groups. Analyzing the HU values of 52 implant sites, the average mandibular bone density was 708.15 ± 182.68 HU, which was significantly higher than maxillary bone density of 622.06 ± 86.93 HU, and the difference was statistically significant (*p* < 0.05) (Table 1). Because of this, the immediate and delayed implantation groups as well as open flap and flapless technique groups were subdivided. The details of the gender, age of subjects and number of implants in different groups were visible in Table 2. There was no statistical difference in gender composition ratio and age between the groups (*p* > 0.05), and the comparability between the groups was good.

### 3.2. Accuracy Analysis

After measurement and statistical calculation, the total apical deviation was 1.13 ± 0.39 mm on average, the total coronal deviation was 0.86 ± 0.33 mm on average, the total depth deviation was 0.41 ± 0.66 mm on average, and the total angle deviation was 3.32 ± 1.65° on average. The statistical results are shown in Table 3.

The mean respective apical deviations (AD) of the maxilla and mandible groups were 1.18 ± 0.41 mm and 1.10 ± 0.38 mm, which meant that the mandible group had smaller apical deviations and better accuracy. On the other hand, in terms of coronal and angular deviations, the two groups showed opposing results. The maxilla group (CD: 0.82 ± 0.32 mm, aD: 3.24 ± 1.33°) was slightly lower than the mandible group (CD: 0.89 ± 0.34 mm, aD: 3.38 ± 1.86°). However, all these differences are not statistically significant (*p* > 0.05). The mean depth deviation (DD) of the maxilla group was 0.64 ± 0.36 mm, and the result of the corresponding group was 0.25 ± 0.77 mm. This result is significant at the *p* = 0.05 level (*p* < 0.05) (Table 4). In this regard, we continued to explore whether the deviation in depth was caused by the different bone density in upper and lower jaws. Pearson product-moment correlation coefficient (Table 5) indicated no significant correlation between bone density and respective deviations. However, the apical deviation positively correlated with coronal, depth, and angular deviations, and so were the coronal deviation and depth deviation. In conclusion, it can be speculated that the different arch type and bone densities would not affect the transferring accuracy of the fully guided implantation in the anterior zone.

**Figure 5:**
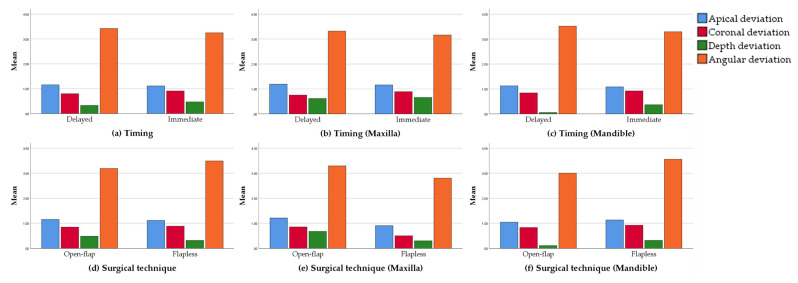
Deviation values of delayed and immediate groups (**a**), maxillary delayed and immediate groups (**b**) and mandible delayed and immediate groups (**c**); deviation values of open flap and flapless groups (**d**), maxillary open flap and flapless groups (**e**) and mandible open flap and flapless groups (**f**).

As shown in Table 6 and Figure 5, the average apical (AD) and angular deviations (aD) of the immediate implantation group were 1.11 ± 0.40 mm and 3.24 ± 1.87°, and the results of the corresponding delayed implantation group were 1.16 ± 0.38 mm and 3.42 ± 1.36°, which means that the former group had higher accuracy. However, the deviations in the shoulder (CD: 0.91 ± 0.3 mm) and depth (DD: 0.47 ± 0.66 mm) of the immediate implantation group were slightly higher than those (CD: 0.80 ± 0.37 mm, DD: 0.33 ± 0.68 mm) of the delayed implantation group. Meanwhile, the magnitude of the difference between the two groups was actually small. Take the arch type with significantly different bone density into consideration, the two groups were subdivided into four. Further analysis of the data reveals the similar tendency of differences between the respective groups with the initial two groups. All differences are not statistically significant (*p* > 0.05). What emerges from the results reported here is that, assisted by the fully guided template, the accuracy of implant placement appeared to be unaffected by the timing of implant placement. However, during the immediate implantation, through the improved preparation sequence, the regulation on deviations in some directions was slightly better than the conventional preparation sequence.

Turning now to the experimental evidence on surgical techniques. The mean apical (AD) and depth deviation (DD) of the open flap technique group were 1.15 ± 0.35 mm and 0.48 ± 0.57 mm. The corresponding results of the flapless technique group were 1.11 ± 0.44 mm and 0.31 ± 0.77 mm, which were slightly lower than those of the open flap technique group. However, the deviations on the shoulder (CD: 0.84 ± 0.34 mm) and angulation (aD: 3.19 ± 1.47°) of the open flap technique group were slightly lower than those (CD: 0.88 ± 0.33 mm, aD: 3.49 ± 1.87°) of the flapless technique group. No statistically significant difference was observed between the two groups (*p* > 0.05). Similarly, we subdivided the groups in line with the arch type. What an interesting outcome is open flap technique applied in mandible acquiring lower measurements in each deviation, whereas the same approach performed in maxilla revealed a completely opposite trend. However, no statistical difference was detected between the respective groups (*p* > 0.05). Hence, it can be stated that the flap elevation did not negatively influence the positioning of the tooth-borne surgical template and that the natural dentition allowed a sufficient anchorage.

## 4. Discussion

Limited by special anatomical locations and some exogenous factors, implantation in the anterior zone is frequently confronted with insufficient bone volume. Compared to posterior area, the available bone width in the anterior zone is thinner. In domestic and abroad investigations, the average thickness of the labial plate in the anterior zone is about 0.7–1.0 m [27,28,29,30]. With the loss of teeth, the labial bundle bone absorbs due to the lack of internal blood supply from periodontal ligament. Thus, the width of the alveolar bone will be further reduced. According to the research of Schropp et al. [31], the alveolar bone width can be absorbed up to 50% within one year after tooth extraction. Fan Shengzi et al. [32] measured the alveolar bone width of the anterior teeth after tooth loss, the results of which showed that after losing teeth for six month, the average bone width 4 mm below the top of the alveolar crest was only 4.10 ± 1.56 mm. One year after the loss, the average bone width was 3.28 ± 0.47 mm.

Performing operations under such limited bone volume conditions, the accuracy of implantation is particularly crucial. A few deviations may induce a series of undesirable consequences. When the implant is placed labially, the implant screw thread may be exposed due to the loss of the labial plate. In that way, additional bone augmentation is required to restore the missing bone during the surgery, which increases the difficulty and complexity of the operation as well as the implant treatment period. In the later stage of restoration, excessively labial inclination may result in later gingival recession, which will compromise the esthetics of the final restoration. When the implant is placed lingually, at the position of the central incisor in maxilla, it may enter into the incisive canal and damage the nasopalatine nerve, inducing paresthesia in the adjacent palatal mucosa. In mandible, the implant may puncture the lingual plate and damage the blood vessels, leading to life-threatening hemorrhage and hematoma [33,34]. Besides, the risk of implant osseointegration failure is increased. In the later stage of restoration, the labial protrusion of the crown is too large for daily oral hygiene maintenance. Whereas the cingulum of the crown is too thick to be comfortable. When the implant is placed mesially or distally, it may hurt adjacent natural teeth. When the distance between the implant and the natural tooth is less than 1.5 mm, the recession of the gingival papilla occurs and will detract the esthetics of the final restoration.

Through computer-assisted, template-guided implant surgery, patients’ information of hard and soft tissues can be visualized in the digital software before the operation. Follow the prosthetic-oriented principle to plan the optimal implant site and keep away from the adjacent anatomical structures. In addition, Koichiro, et al. revealed that the optimal implant position can also be determined through biomechanical considerations coming from stress analysis [35]. Under the guidance of the fully guided template, the optimal three-dimensional position of the implant can come true in the anterior zone. The application of the template ensures a high positional precision, and effectively utilizes the available bone volume and reduces unnecessary bone loss. At the same time, it makes the operation more minimally invasive, increases the patients’ comfort and shortens the surgical time.

In this study, the apical, coronal, depth and angular deviations between the actual implant position and the planned position was 1.13 ± 0.39 mm, 0.86 ± 0.33 mm, 0.41 ± 0.66 mm and 3.32 ± 1.65° on average. These results are consistent with the findings of Dreiseidler, Van de Wiele, D’Haese. Dreiseidler et al. [36] carried out an in vitro experiment with a total of 108 implants were inserted in partially edentulous models. The average deviations at shoulder level and apex level were 0.89 ± 0.44 mm and 1.09 ± 0.69 mm, respectively. Van de Wiele [37] and D’Haese [38] measured 75 and 77 implants placed in edentulous patients. The mean coronal deviations were 0.88 ± 0.50 mm and 0.91 ± 0.44 mm, and the mean apical deviations corresponded to 1.10 ± 0.53 mm and 1.13 ± 0.52 mm.

In some other reports, the average deviations of the fully guided template at the shoulder and apex can be as low as 0.32 ± 0.23 mm, 0.49 ± 0.29 mm [39], and as high as 1.96 ± 0.23 mm, 2.29 ± 0.27 mm [40]. One of the reasons for the inconsistency of these results may be the divergence in research models. Generally, in the case of in vitro experiments and cadaveric studies, better visibility makes the operation easier. However, in clinical situations, clinicians are usually faced with more complicated oral conditions, which may affect the final accuracy. On the other hand, different measurement methods will also contribute to different outcomes. For example, some researchers measured the linear distance of the apical and coronal center between the virtual planned implant and the actual implant. While others measure the horizontal distance between the two implants at shoulder level and apex level. So far, there is no standardized index to measure the accuracy of implant placement. Diverse methods come to disparate results, which makes different studies less comparable.

Although there are certain differences in the transferring accuracy of the fully guided template among different research studies, more and more studies believe that the overall accuracy of the fully guided surgery is better than that of the pilot-drill-guided and free-hand surgery. A retrospective study by Cassetta et al. [13] pointed out that the fully guided template can provide better accuracy at shoulder level and depth than the pilot-drill-guided. At the 4th EAO Consensus Conference, the point of view that fully guided surgery has higher accuracy has been repeatedly raised [41]. Furthermore, recently, Fernando et al. [16] conducted a systematic review of computer-guided surgery and compared the accuracy of the pilot-drill-guided template and the fully guided template. The coronal, apical, depth and angular deviations of the full-course guide were 1.00 ± 0.08 mm, 1.23 ± 0.10 mm, 0.62 ± 0.08 mm, 3.13 ± 0.23°, which are similar to the results of this study. In contrast, the deviation of the half-course guide at each position has been increased, corresponding to 1.44 ± 0.18 mm, 1.91 ± 0.23 mm, 0.83 ± 0.23 mm, 4.30 ± 0.73°. The transferring accuracy of pilot-drill-guided template is worse than the fully guided template.

There is still some controversy about the influence of the implant site, that is, the maxilla and mandible on the accuracy of fully guided implantation. Some researchers argued that the accuracy of the maxillary guided template is higher than the mandibular one. For example, Lin [42] and Behneke [26] showed that the deviations at apex level of the mandibular guided template are significantly lower than those of the maxillary one, while Vasak et al. [43] thought that the difference is mainly manifested at shoulder level. Christache et al. [44] revealed that the coronal, apical and depth deviations of the mandibular guided implantation are smaller than those of the maxillary implantation. In this regard, the researchers analyzing the reasons for the difference may be related to the bone density of the jaw. The bone density of the upper jaw is lower than that of the lower jaw. The drill faced with less resistance is easier to deviate during preparation and implantation [39]. However, some scholars hold different opinions. Valent [3] and Erosy [45] showed that the accuracy of the mandibular guided template is higher than that of the maxillary one, because the upper jaw provides a large-scale support to the guided template to obtain good stability [46]. Zhou et al. [47] conducted a systematic review of the factors that affect the accuracy of guided surgery, and analyzed that the maxillary and mandibular guided operations have no significant difference in the linear deviations, but the angular deviation. In our study, except that the depth deviation in maxilla group was slightly higher than that in mandible group, and the differences in the other three deviations were not statistically significant.

Analyze the possible reasons. First, tooth-supported template with retention of guided anchor pin determines good stability in the upper and lower jaws. The second is that the anterior zone supplies strong operability on better vision, easy operation, less interference from factors such as mouth opening, which is different from the posterior tooth area. Third, the entire surgical process from initial positioning to final implant placement is assisted by the guided template, which relatively weakens the interference of some unfavorable factors such as jaw bone density. Therefore, the accuracy appeared to be unaffected by the arch type and the bone density. The significant difference on depth deviation in this study may be caused by the surgeon’s habit of using a torque wrench to deepen one or two screw threads of the implant after the guided template is removed from the upper jaw.

Unlike conventional implantation, immediate implantation is to do the implant site preparation in the empty extraction socket. During the preparation process, the resistance from lingual side is greater than that from buccal side due to the existence of the lingual bone wall, so the drill easily deviates to the buccal side, which affects the accuracy of the implantation. Although the implant can be placed in a more optimal position under the guidance of the guided template, the drill may still slip and drift during preparation. Because it is impossible to restrict the drill in whole length due to the limited height of the guide sleeve, and there are tolerances between the guide sleeve and the drill. In this study, the accuracy of immediate implantation is similar to that of delayed implantation. And in terms of apical and angular deviations, immediate implantation group appeared higher precision, which is accordant to the results of Alzoubi et al. [48], who compared the deviations in the three directions of shoulder level, apex level and angulation. Into the immediate implantation cases discrepancies of shoulder level, apex level and angulation were observed as 0.85 mm, 1.10 mm and 3.49°, respectively, while delayed implantation group corresponds to 0.88 mm, 1.59 mm and 4.29°, which are lower than the former group. It can be observed that by removing the resistance of the lingual bone wall in advance, and cooperating with the fully guided template, immediate implantation and conventional implantation can achieve similar accuracy. In this study, the average implant depth of the immediate implantation group was slightly deeper than that of the conventional implant group. The possible reason was that in this research group immediate load was usually performed during the operation, which required axial adjustment of the implant according to the corresponding marking points on the guided template, so that the temporary prosthesis could be accurately positioned, and the implant depth was deepened during this process.

Conventional implant surgery needs to elevate flaps, expose the implant site, and suture to seal the wounds, which have been obtained satisfactory healing and restoration. However, the open flap will reduce the blood supply of the periosteum, leading to the loss of alveolar bone mass, and postoperative bleeding, increasing the risk of infection and gingival recession [49]. The flapless implant surgery assisted by surgical template can prevent from the adverse effects of conventional implant surgery, that is, reducing the patient’s swelling and pain, intraoperative bleeding and operation time, without sutures, and preserving the soft and hard tissues of the implant site as well as maintaining blood supply so that patients can return to normal oral hygiene as soon as possible [50]. However, flapless surgery is appropriate for sufficient vertical height, width and bone density of the alveolar bone, at least 3 mm attached gum and at least 50 mm extent of mouth opening [51].

Among the 52 implants in this study, there was no statistically significant difference in the measurement indexes between the open flap technique group and the flapless technique group. This is consistent with the reports of Schnutenhaus et al. [25] and Ersoy et al. [45] Most of the parameters in the study of Behneke et al. [26] did not differ significantly during surgery. The only significant difference (*p* = 0.027) is that the implant position of the flapless technique group is shallower than the actual implant position of the open flap technique group. In our study, the average implant depth of the flapless technique group was slightly shallower than that of the open flap technique group, but the difference was not statistically significant. A systematic review by Tahmaseb et al. [52] pointed out that in clinical studies, the accuracy of open flap technique group is much lower than that of flapless technique group, because most open flap are performed under the guidance of bone-borne templates. Van Assche [16] conducted a meta-analysis and found that bone-borne templates affect the accuracy of digital guided surgical. However, the angular and apical deviations of the tooth-borne guided template are significantly smaller than the mucosa-borne and bone-borne methods [20]. It can be indicated that under the guidance of the tooth-borne, fully guided template, the surgical method does not affect the accuracy of implant placement.

Studies have shown that compared to the surgical stage, more deviations are related with the preoperative stage [39]. In the process of preoperative data collection, although CBCT is currently a relatively reliable imaging method, factors such as metal restorations in the mouth and shaking of the patient during the shooting process may cause the final image to be distorted and inaccurate, which results in bias in the overlap and evaluation of the data [53,54]. In the process of oral scanning to collect information in the patient’s mouth, blood, saliva, the size of the scanning head, and improper operation methods may cause deviations in the final data [55,56]. In addition, the accuracy of the optical scanner and the digital design software will also affect the final precision [57,58]. At the stage of implant surgery, there is a certain tolerance between the guide sleeve and the drill. When the drill is drilling, there may be a certain extent of movement between them, which allows the preparation direction of the implant somewhat changed. Some scholars believe that by reducing the height from bottom of the guide sleeve to the bone surface and increasing the length of guide sleeve, the deviation can be reduced, and the accuracy can be improved [59,60]. The NobelGuide^®^ fully guided template and the matching kit used in this study have certain tolerances, although it can allow the surgeon to better perceive the implant placement, it may also have a certain impact on the accuracy of implant placement. Furthermore, in a recent study, Cassetta et al. [61] found that the experience of the surgeon had almost no effect on the guided surgery.

This study merely focused on the transferring accuracy of the NobelGuide^®^ templates in the anterior zone, and lacked the comparison of other systems, the conclusions of which had certain one-sidedness and limitations, and was unable to encompass the results of entire guided templates. Furthermore, as a retrospective cohort study, it only reported 40 patients, a total of 52 implants, the sample size of which was too insufficient to provide a strong evidence-based argument. Therefore, relevant randomized controlled trials and large numbers of samples are urgently needed to give further verifications of the findings.

## 5. Conclusions

In the anterior zone, there are certain deviations at shoulder level, apex level, depth and angulation between the position of the implant guided by the fully guided template and the preoperative planned position. However, compared to the data of the previous literature, its transferring accuracy is higher than the pilot-drill-guided template. Thus, tooth-borne, fully guided templates can guarantee a relatively high transferring accuracy of implantation. The implant site, alveolar bone density, timing of implant placement and surgical techniques hardly ever compromise the accuracy.

## Figures and Tables

**Table 1:** *t*-test specific to the influence of arch type on the bone density (N = 52).

	Maxilla	Mandible	*p* Value
Bone density (HU)	622.06 ± 86.93	708.15 ± 182.68	0.028 *

* Significance was *p* < 0.05.

**Table 2:** Patients and treatment characteristics.

		No. Implants		Gender		Age	
		Maxilla	Mandible	Male	Female	Mean ± SD	Range
Total		52		17	23	44.40 ± 14.43	18–75
Arch	Maxilla	21		6	14	42.10 ± 10.98	26–63
	Mandible		31	11	9	46.70 ± 17.20	18–75
Timing	Immediate	10	19	10	12	45.59 ± 14.99	18–75
	Delayed	11	12	7	11	42.94 ± 14.01	18–68
Surgical technique	Open flap	19	10	10	15	44.68 ± 12.27	18–68
	Flapless	2	21	7	8	43.93 ± 17.93	18–75

**Table 3:** Accuracy: deviation values.

	Mean	SD	Max.	Min.
Apical deviation (mm)	1.13	0.39	1.80	0.40
Coronal deviation (mm)	0.86	0.33	1.60	0.10
Depth deviation (mm)	0.41	0.66	1.50	0.00
Angular deviation (°)	3.32	1.65	8.60	0.40

**Table 4:** *t*-test specific to the influence of arch type on the bone density and the deviation values (N = 52).

	Maxilla	Mandible	*p* Value
Apical deviation (mm)	1.18 ± 0.41	1.10 ± 0.38	0.493
Coronal deviation (mm)	0.82 ± 0.32	0.89 ± 0.34	0.455
Depth deviation (mm)	0.64 ± 0.36	0.25 ± 0.77	0.036 *
Angular deviation (deg)	3.24 ± 1.33	3.38 ± 1.86	0.776

* Significance was *p* < 0.05.

**Table 5:** Correlation between the bone density and the different deviation parameters. (*p* value) (N = 52).

		Apical Deviation	Coronal Deviation	Depth Deviation	Angular Deviation
Bone density	Pearson correlation	−0.006	0.119	−0.164	0.004
	Sig.	0.967	0.403	0.245	0.976
	N	52	52	52	52
Angular deviation	Pearson correlation	0.490 **	0.220	−0.153	
	Sig.	0.000	0.118	0.280	
	N	52	52	52	
Depth deviation	Pearson correlation	0.311 **	0.474 **		
	Sig.	0.025	0.000		
	N	52	52		
Coronal deviation	Pearson correlation	0.544 **			
	Sig.	0.000			
	N	52			

** Significance was *p* < 0.01.

**Table 6:** *t*-Test specific to the influence of timing of implant placement and surgical technique on the deviation values (N = 52).

		Apical Deviation (mm)		Coronal Deviation (mm)		Depth Deviation (mm)		Angular Deviation (deg)	
		Mean ± SD	*p* Value	Mean ± SD	*p* Value	Mean ± SD	*p* Value	Mean ± SD	*p* Value
Immediate		1.11 ± 0.40	0.675	0.91 ± 0.30	0.239	0.47 ± 0.66	0.445	3.24 ± 1.87	0.705
Delayed		1.16 ± 0.38		0.80 ± 0.37		0.33 ± 0.68		3.42 ± 1.36	
Maxilla	Immediate	1.16 ± 0.39	0.868	0.89 ± 0.27	0.353	0.66 ± 0.37	0.800	3.16 ± 1.35	0.793
	Delayed	1.19 ± 0.45		0.75 ± 0.28		0.62 ± 0.38		3.32 ± 1.37	
Mandible	Immediate	1.08 ± 0.42	0.775	0.92 ± 0.27	0.537	0.37 ± 0.76	0.283	3.29 ± 2.13	0.746
	Delayed	1.13 ± 0.31		0.84 ± 0.44		0.06 ± 0.79		3.52 ± 1.41	
Open flap		1.15 ± 0.35	0.719	0.84 ± 0.34	0.689	0.48 ± 0.57	0.374	3.19 ± 1.47	0.518
Flapless		1.11 ± 0.44		0.88 ± 0.33		0.31 ± 0.77		3.49 ± 1.87	
Maxilla	Open flap	1.21 ± 0.40	0.329	0.85 ± 0.31	0.149	0.67 ± 0.34	0.173	3.29 ± 1.35	0.633
	Flapless	0.90 ± 0.57		0.50 ± 0.42		0.30 ± 0.57		2.80 ± 1.41	
Mandible	Open flap	1.04 ± 0.20	0.444	0.83 ± 0.41	0.506	0.11 ± 0.73	0.500	3.00 ± 1.74	0.444
	Flapless	1.13 ± 0.44		0.92 ± 0.31		0.31 ± 0.80		3.56 ± 1.92	

## Data Availability

Data is contained within the article or Supplementary Materials.

## Supplementary Materials

The following are available online at https://www.mdpi.com/article/10.3390/ma14164631/s1, Table S1: Initial data of patients’ characteristics and treatment.

## References

[1] Buser D., Martin W., Belser U.C. (2004). Optimizing esthetics for implant restorations in the anterior maxilla: Anatomic and surgical considerations. Int. J. Oral Maxillofac. Implant..

[2] Ozan O., Turkyilmaz I., Ersoy A.E., McGlumphy E.A., Rosenstiel S.F. (2009). Clinical accuracy of 3 different types of computed tomography-derived stereolithographic surgical guides in implant placement. J. Oral Maxillofac. Surg..

[3] Valente F., Schiroli G., Sbrenna A. (2009). Accuracy of computer-aided oral implant surgery: A clinical and radiographic study. Int. J. Oral Maxillofac. Implant..

[4] Arisan V., Karabuda Z.C., Ozdemir T. (2010). Accuracy of two stereolithographic guide systems for computer-aided implant placement: A computed tomography-based clinical comparative study. J. Periodontol..

[5] Becker C.M., Kaiser D.A. (2000). Surgical guide for dental implant placement. J. Prosthet. Dent..

[6] Almog D.M., Torrado E., Meitner S.W. (2001). Fabrication of imaging and surgical guides for dental implants. J. Prosthet. Dent..

[7] Katsoulis J., Pazera P., Mericske-Stern R. (2009). Prosthetically driven, computer-guided implant planning for the edentulous maxilla: A model study. Clin. Implant. Dent. Relat. Res..

[8] Chen S., Ou Q., Lin X., Wang Y. (2019). Comparison between a computer-aided surgical template and the free-hand method: A systematic review and meta-analysis. Implant. Dent..

[9] Chen Z., Li J., Sinjab K., Mendonca G., Yu H., Wang H.L. (2018). Accuracy of flapless immediate implant placement in anterior maxilla using computer-assisted versus freehand surgery: A cadaver study. Clin. Oral Implant. Res..

[10] Younes F., Cosyn J., de Bruyckere T., Cleymaet R., Bouckaert E., Eghbali A. (2018). A randomized controlled study on the accuracy of free-handed, pilot-drill guided and fully guided implant surgery in partially edentulous patients. J. Clin. Periodontol..

[11] Vermeulen J. (2017). The accuracy of implant placement by experienced surgeons: Guided vs. freehand approach in a simulated plastic model. Int. J. Oral Maxillofac. Implant..

[12] Tallarico M., Park C.J., Lumbau A.I., Annucci M., Baldoni E., Koshovari A., Meloni S.M. (2020). Customized 3D-printed titanium mesh developed to regenerate a complex bone defect in the aesthetic zone: A case report approached with a fully digital workflow. Materials.

[13] Cassetta M., Giansanti M., Di Mambro A., Calasso S., Barbato E. (2013). Accuracy of two stereolithographic surgical templates: A retrospective study. Clin. Implant Dent. Relat. Res..

[14] Azari A., Nikzad S. (2008). Computer-assisted implantology: Historical background and potential outcomes—A review. Int. J. Med. Robot..

[15] Chen X., Xu L., Wang W., Li X., Sun Y., Politis C. (2016). Computer-aided design and manufacturing of surgical templates and their clinical applications: A review. Expert Rev. Med. Devices.

[16] Bover-Ramos F., Vina-Almunia J., Cervera-Ballester J., Penarrocha-Diago M., Garcia-Mira B. (2018). Accuracy of implant placement with computer-guided surgery: A systematic review and meta-analysis comparing cadaver, clinical, and in vitro studies. Int. J. Oral Maxillofac. Implant..

[17] Kuhl S., Zurcher S., Mahid T., Muller-Gerbl M., Filippi A., Cattin P. (2013). Accuracy of full guided vs. half-guided implant surgery. Clin. Oral Implant. Res..

[18] Van Assche N., Vercruyssen M., Coucke W., Teughels W., Jacobs R., Quirynen M. (2012). Accuracy of computer-aided implant placement. Clin. Oral Implant. Res..

[19] Beretta M., Poli P.P., Maiorana C. (2014). Accuracy of computer-aided template-guided oral implant placement: A prospective clinical study. J. Periodontal Implant Sci..

[20] Skjerven H., Riis U.H., Herlofsson B.B., Ellingsen J.E. (2019). In Vivo Accuracy of Implant Placement Using a Full Digital Planning Modality and Stereolithographic Guides. Int. J. Oral Maxillofac. Implant..

[21] Ozan O., Orhan K., Turkyilmaz I. (2011). Correlation between bone density and angular deviation of implants placed using CT-generated surgical guides. J. Craniofacial Surg..

[22] Del Fabbro M., Testori T., Kekovic V., Goker F., Tumedei M., Wang H.L. (2019). A systematic review of survival rates of osseointegrated implants in fully and partially edentulous patients following immediate loading. J. Clin. Med..

[23] Chrcanovic B.R., Albrektsson T., Wennerberg A. (2014). Immediate nonfunctional versus immediate functional loading and dental implant failure rates: A systematic review and meta-analysis. J. Dent..

[24] Albrektsson T., Buser D., Chen S.T., Cochran D., DeBruyn H., Jemt T., Koka S., Nevins M., Sennerby L., Simion M. (2012). Statements from the Estepona consensus meeting on peri-implantitis, 2–4 February 2012. Clin. Implant Dent. Relat. Res..

[25] Schnutenhaus S., Edelmann C., Rudolph H., Dreyhaupt J., Luthardt R.G. (2018). 3D accuracy of implant positions in template-guided implant placement as a function of the remaining teeth and the surgical procedure: A retrospective study. Clin. Oral Investig..

[26] Behneke A., Burwinkel M., Behneke N. (2012). Factors influencing transfer accuracy of cone beam CT-derived template-based implant placement. Clin. Oral Implant. Res..

[27] Huynh-Ba G., Pjetursson B.E., Sanz M., Cecchinato D., Ferrus J., Lindhe J., Lang N.P. (2010). Analysis of the socket bone wall dimensions in the upper maxilla in relation to immediate implant placement. Clin. Oral Implant. Res..

[28] Xiaoshi J., Qiuxia Z., Mengjie X., Meng W., Peng Z. (2015). The labial and palatal bone thickness in 67 young adults with normal occlusion at the maxillary anterior teeth measured by cone-beam computed tomography. Shanghai J. Stomatol..

[29] Vera C., de Kok I.J., Reinhold D., Limpiphipatanakorn P., Yap A.K., Tyndall D., Cooper L.F. (2012). Evaluation of buccal alveolar bone dimension of maxillary anterior and premolar teeth: A cone beam computed tomography investigation. Int. J. Oral Maxillofac. Implant..

[30] Xiao Z., Yuehua L. (2013). Evaluation of labio-lingual alveolar bone thickness of anterior incisors in normal occlusion of young adult. J. Clin. Stomatol..

[31] Schropp L., Wenzel A., Kostopoulos L., Karring T. (2003). Bone healing and soft tissue contour changes following single-tooth extraction: A clinical and radiographic 12-month prospective study. Int. J. Periodontics Restor. Dent..

[32] Shengzi F. (2016). A Study on Thickness of Labial Bone Plates and Gingival Tissue in Maxillary Anterior Teeth by Cone Beam Computed Tomography. Master’s Thesis.

[33] Law C., Alam P., Borumandi F. (2017). Floor-of-mouth hematoma following dental implant placement: Literature review and case presentation. J. Oral Maxillofac. Surg. Off. J. Am. Assoc. Oral Maxillofac. Surg..

[34] Kalpidis C.D., Setayesh R.M. (2004). Hemorrhaging associated with endosseous implant placement in the anterior mandible: A review of the literature. J. Periodontol..

[35] Montero J., Fernández-Ruiz A., Pardal-Peláez B., Jiménez-Guerra A., Velasco-Ortega E., Nicolás-Silvente A.I., Monsalve-Guil L. (2020). Effect of rough surface platforms on the mucosal attachment and the marginal bone loss of implants: A dog study. Materials.

[36] Dreiseidler T., Tandon D., Kreppel M., Neugebauer J., Mischkowski R.A., Zinser M.J., Zoller J.E. (2012). CBCT device dependency on the transfer accuracy from computer-aided implantology procedures. Clin. Oral Implant. Res..

[37] Van de Wiele G., Teughels W., Vercruyssen M., Coucke W., Temmerman A., Quirynen M. (2015). The accuracy of guided surgery via mucosa-supported stereolithographic surgical templates in the hands of surgeons with little experience. Clin. Oral Implant. Res..

[38] D’Haese J., van de Velde T., Elaut L., de Bruyn H. (2012). A prospective study on the accuracy of mucosally supported stereolithographic surgical guides in fully edentulous maxillae. Clin. Implant. Dent. Relat. Res..

[39] Behneke A., Burwinkel M., Knierim K., Behneke N. (2012). Accuracy assessment of cone beam computed tomography-derived laboratory-based surgical templates on partially edentulous patients. Clin. Oral Implant. Res..

[40] Verhamme L.M., Meijer G.J., Berge S.J., Soehardi R.A., Xi T., de Haan A.F., Schutyser F., Maal T.J. (2015). An accuracy study of computer-planned implant placement in the augmented maxilla using mucosa-supported surgical templates. Clin. Implant. Dent. Relat. Res..

[41] Hammerle C.H., Cordaro L., van Assche N., Benic G.I., Bornstein M., Gamper F., Gotfredsen K., Harris D., Hurzeler M., Jacobs R. (2015). Digital technologies to support planning, treatment, and fabrication processes and outcome assessments in implant dentistry. Summary and consensus statements. The 4th EAO consensus conference 2015. Clin. Oral Implant. Res..

[42] Lin Y.K., Yau H.T., Wang I.C., Zheng C., Chung K.H. (2015). A novel dental implant guided surgery based on integration of surgical template and augmented reality. Clin. Implant. Dent. Relat. Res..

[43] Vasak C., Watzak G., Gahleitner A., Strbac G., Schemper M., Zechner W. (2011). Computed tomography-based evaluation of template (NobelGuide)-guided implant positions: A prospective radiological study. Clin. Oral Implant. Res..

[44] Cristache C.M., Gurbanescu S. (2017). Accuracy evaluation of a stereolithographic surgical template for dental implant insertion using 3D superimposition protocol. Int. J. Dent..

[45] Ersoy A.E., Turkyilmaz I., Ozan O., McGlumphy E.A. (2008). Reliability of implant placement with stereolithographic surgical guides generated from computed tomography: Clinical data from 94 implants. J. Periodontol..

[46] Vercruyssen M., Cox C., Coucke W., Naert I., Jacobs R., Quirynen M. (2014). A randomized clinical trial comparing guided implant surgery (bone- or mucosa-supported) with mental navigation or the use of a pilot-drill template. J. Clin. Periodontol..

[47] Zhou W., Liu Z., Song L., Kuo C.L., Shafer D.M. (2018). Clinical factors affecting the accuracy of guided implant surgery—A systematic review and meta-analysis. J. Evid. Based Dent. Pract..

[48] Alzoubi F., Massoomi N., Nattestad A. (2016). Accuracy assessment of immediate and delayed implant placements using CAD/CAM surgical guides. J. Oral Implantol..

[49] Rao J.B., Tatuskar P., Pulla A., Kumar N., Patil S.C., Tiwari I. (2018). Radiographic assessment of anatomy of nasopalatine canal for dental implant placement: A cone beam computed tomographic study. J. Contemp. Dent. Pract..

[50] Yeung M., Abdulmajeed A., Carrico C.K., Deeb G.R., Bencharit S. (2020). Accuracy and precision of 3D-printed implant surgical guides with different implant systems: An in vitro study. J. Prosthet. Dent..

[51] de Almeida E.O., Pellizzer E.P., Goiatto M.C., Margonar R., Rocha E.P., Freitas A.C., Anchieta R.B. (2010). Computer-guided surgery in implantology: Review of basic concepts. J. Craniofacial Surg..

[52] Tahmaseb A., Wismeijer D., Coucke W., Derksen W. (2014). Computer technology applications in surgical implant dentistry: A systematic review. Int. J. Oral Maxillofac. Implant..

[53] Tadinada A., Jalali E., Jadhav A., Schincaglia G.P., Yadav S. (2015). Artifacts in cone beam computed tomography image volumes: An illustrative depiction. J. Mass. Dent. Soc..

[54] Pettersson A., Komiyama A., Hultin M., Nasstrom K., Klinge B. (2012). Accuracy of virtually planned and template guided implant surgery on edentate patients. Clin. Implant. Dent. Relat. Res..

[55] Gimenez B., Pradies G., Martinez-Rus F., Ozcan M. (2015). Accuracy of two digital implant impression systems based on confocal microscopy with variations in customized software and clinical parameters. Int. J. Oral Maxillofac. Implant..

[56] Gimenez B., Ozcan M., Martinez-Rus F., Pradies G. (2015). Accuracy of a digital impression system based on active wavefront sampling technology for implants considering operator experience, implant angulation, and depth. Clin. Implant Dent. Relat. Res..

[57] Tahmaseb A., Wu V., Wismeijer D., Coucke W., Evans C. (2018). The accuracy of static computer-aided implant surgery: A systematic review and meta-analysis. Clin. Oral Implant. Res..

[58] Im C.H., Park J.M., Kim J.H., Kang Y.J., Kim J.H. (2020). Assessment of compatibility between various intraoral scanners and 3D printers through an accuracy analysis of 3D printed models. Materials.

[59] D’Haese J., van de Velde T., Komiyama A., Hultin M., de Bruyn H. (2012). Accuracy and complications using computer-designed stereolithographic surgical guides for oral rehabilitation by means of dental implants: A review of the literature. Clin. Implant Dent. Relat. Res..

[60] Koop R., Vercruyssen M., Vermeulen K., Quirynen M. (2013). Tolerance within the sleeve inserts of different surgical guides for guided implant surgery. Clin. Oral Implant. Res..

[61] Cassetta M., Altieri F., Giansanti M., Bellardini M., Brandetti G., Piccoli L. (2020). Is there a learning curve in static computer-assisted implant surgery? A prospective clinical study. Int. J. Oral Maxillofac. Surg..

