# Supplementary material for: A Retrospective Study on the Transferring Accuracy of a Fully Guided Digital Template in the Anterior Zone
Source: Materials (Basel). 2021 Aug 17;14(16):4631. doi: 10.3390/ma14164631 (PMC8399113; doi:10.3390/ma14164631)
Supplement: Supplementary file 1 [file materials-14-04631-s001.zip › materials-1222919-supplementary.pdf]

materials

Supplementary

A Retrospective Study on the Transferring Accuracy of a Fully Guided Digital Template in the Anterior Zone

Lirong Huang, Xiaoqing Zhang and Anchun Mo

Table S1. Initial data of patients' characteristics and treatment.

No.	Gender	Age	Implant site	Arch	Timing	Surgical technique	Bone density
1	Female	26	21	Maxilla	Delayed	Open-flap	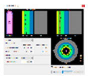
2	Male	55	12	Maxilla	Immediate	Open-flap	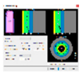
			11	Maxilla	Delayed	Open-flap	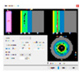
3	Male	63	22	Maxilla	Immediate	Open-flap	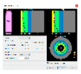
4	Female	32	11	Maxilla	Immediate	Open-flap	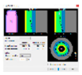
5	Female	33	22	Maxilla	Delayed	Open-flap	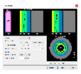
6	Female	41	11	Maxilla	Immediate	Open-flap	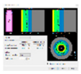
7	Female	51	21	Maxilla	Immediate	Open-flap	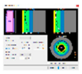
8	Female	45	21	Maxilla	Immediate	Open-flap	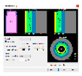
9	Female	30	11	Maxilla	Delayed	Open-flap	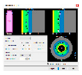
10	Male	39	21	Maxilla	Immediate	Open-flap	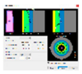
11	Female	28	11	Maxilla	Immediate	Open-flap	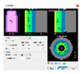
12	Male	48	21	Maxilla	Delayed	Open-flap	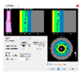
13	Female	52	21	Maxilla	Immediate	Open-flap	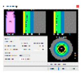
14	Female	32	21	Maxilla	Immediate	Flapless	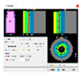

15	Female	31	23	Maxilla	Delayed	Flapless	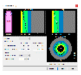
16	Male	50	22	Maxilla	Delayed	Open-flap	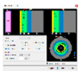
17	Male	49	21	Maxilla	Delayed	Open-flap	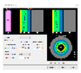
18	Female	55	21	Maxilla	Delayed	Open-flap	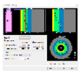
19	Female	50	21	Maxilla	Delayed	Open-flap	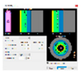
20	Female	32	13	Maxilla	Delayed	Open-flap	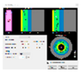
21	Male	52	32	Mandible	Delayed	Open-flap	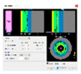
			42	Mandible	Delayed	Open-flap	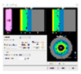
22	Female	18	41	Mandible	Immediate	Flapless	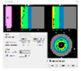
23	Male	21	41	Mandible	Delayed	Flapless	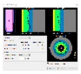
24	Female	49	41	Mandible	Immediate	Flapless	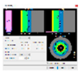
			32	Mandible	Delayed	Flapless	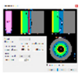
25	Female	52	32	Mandible	Delayed	Flapless	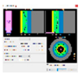
26	Female	21	43	Mandible	Immediate	Flapless	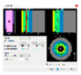
27	Female	55	42	Mandible	Immediate	Flapless	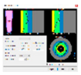
			32	Mandible	Immediate	Flapless	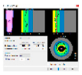
28	Male	42	32	Mandible	Immediate	Open-flap	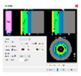
			42	Mandible	Immediate	Open-flap	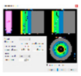
29	Male	50	32	Mandible	Immediate	Flapless	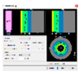
			42	Mandible	Immediate	Flapless	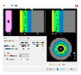
30	Male	59	31	Mandible	Immediate	Flapless	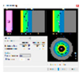

			42	Mandible	Immediate	Flapless	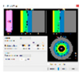
31	Female	48	31	Mandible	Delayed	Open-flap	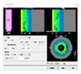
32	Male	68	31	Mandible	Delayed	Open-flap	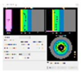
33	Male	75	32	Mandible	Immediate	Flapless	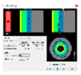
			42	Mandible	Immediate	Flapless	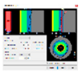
34	Male	59	32	Mandible	Immediate	Flapless	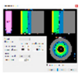
			42	Mandible	Immediate	Flapless	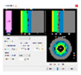
35	Female	55	32	Mandible	Delayed	Open-flap	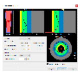
			42	Mandible	Delayed	Open-flap	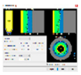
36	Female	55	41	Mandible	Delayed	Open-flap	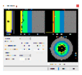
37	Male	18	41	Mandible	Delayed	Open-flap	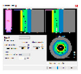
38	Female	63	31	Mandible	Immediate	Flapless	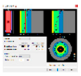
			42	Mandible	Immediate	Flapless	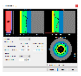
39	Male	24	33	Mandible	Immediate	Flapless	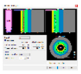
40	Male	50	31	Mandible	Immediate	Flapless	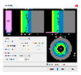
			42	Mandible	Delayed	Flapless	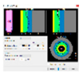
